# Driving Oscillatory Dynamics: Neuromodulation for Recovery After Stroke

**DOI:** 10.3389/fnsys.2021.712664

**Published:** 2021-07-22

**Authors:** Sven Storch, Montana Samantzis, Matilde Balbi

**Affiliations:** Queensland Brain Institute, The University of Queensland, Brisbane, QLD, Australia

**Keywords:** stroke, neuromodulation, brain oscillations, neuroprotection, optical technologies

## Abstract

Stroke is a leading cause of death and disability worldwide, with limited treatments being available. However, advances in optic methods in neuroscience are providing new insights into the damaged brain and potential avenues for recovery. Direct brain stimulation has revealed close associations between mental states and neuroprotective processes in health and disease, and activity-dependent calcium indicators are being used to decode brain dynamics to understand the mechanisms underlying these associations. Evoked neural oscillations have recently shown the ability to restore and maintain intrinsic homeostatic processes in the brain and could be rapidly deployed during emergency care or shortly after admission into the clinic, making them a promising, non-invasive therapeutic option. We present an overview of the most relevant descriptions of brain injury after stroke, with a focus on disruptions to neural oscillations. We discuss the optical technologies that are currently used and lay out a roadmap for future studies needed to inform the next generation of strategies to promote functional recovery after stroke.

## Introduction

Stroke is a debilitating neurological condition that constitutes a major cause of adult disability, affecting 10 million patients annually. Recent advances in treatment have improved the prognosis of stroke survivors, but few treatment options are available for most patients. Tissue plasminogen activator (tPA), the gold standard treatment for ischemic stroke, can break up the clot if administered within a narrow therapeutic window of <4.5 h (Cheatwood et al., 2008). However, <5% of patients are eligible to be treated with tPA (Henninger and Fisher, 2016), requiring a new strategic approach to guide translational interventions.

Following stroke changes occur at the molecular, circuit, and behavioural levels. These include activation of inflammatory pathways and increased oxidative stress (Moskowitz et al., 2010). On a circuit and interhemispheric level, there is an imbalance of inhibitory and excitatory neuronal activity, and disruption of neural networks (Aronowski and Zhao, 2011). Ultimately, these changes lead to neuronal death and loss of synaptic connections that, depending on which part of the brain is affected, result in behavioural deficits such as weakness, limb hemiparesis, and loss of coordination (Hatem et al., 2016; Lodha et al., 2017), as well as speech and cognitive impairments (Sun et al., 2014). This loss of function can be partly recovered due to neuroplastic processes, including the rewiring of neural connections and compensation from other brain regions (Alia et al., 2017). The peri-infarct area is the major region where this plasticity occurs, through the expression of both growth-promoting and growth-inhibitory proteins that induce key neural plasticity processes including spinogenesis, and intense rewiring of neuronal circuits (Carmichael, 2006; Overman et al., 2012; Clarkson et al., 2013; Silasi and Murphy, 2014). Researchers have harnessed these neuroplastic processes to promote recovery in stroke survivors by using neuromodulatory pharmaceuticals and stimulation techniques including exercise, GABA_A_ receptor antagonists, and brain stimulation (Boddington and Reynolds, 2017; Caglayan et al., 2019; Inoue et al., 2020).

Brain stimulation methods are currently used in the treatment of many disorders, including obsessive compulsive disorder, depression, and epilepsy (Johnson et al., 2013). Invasive and non-invasive stimulation has led to promising motor recovery in several disorders such as Parkinson's disease (PD), tremors, and spinal cord injuries (Johnson et al., 2013). Deep brain stimulation (DBS) is a method of invasive stimulation used to treat stroke (Elias et al., 2018), while non-invasive approaches include transcranial magnetic stimulation (TMS) (Smith and Stinear, 2016), transcranial direct current stimulation (tDCS) (Sawan et al., 2020), and transcranial alternating current stimulation (tACS) (Solomons and Shanmugasundaram, 2019). These techniques rely on different electromagnetic principles to modulate brain activity, and their effects which range from the molecular to the behavioural level, are still poorly understood. Changes to resting oscillatory brain activity are key features in several neurological disorders (Başar et al., 2016; Assenza et al., 2017), leading Krawinkel et al. to suggest that brain stimulation tools could be used to modulate abnormal oscillatory activity and guide behavioural recovery (Krawinkel et al., 2015). This type of targeted neuromodulation has since shown promising effects in the treatment of PD, Alzheimer's disease (AD) and epilepsy, among other neurological disorders (Andrade et al., 2016; Mably and Colgin, 2018).

In this review we focus our attention on the recent advances in stroke recovery related to changes in brain oscillations. We present an overview on how brain stimulation techniques drive neural oscillations and lay out a roadmap for future studies that are needed to inform the next generation of strategies to promote functional recovery after stroke.

## Oscillatory Activity In the Brain

Brain oscillations are rhythmic patterns of neuronal firing generated by the synchronised interaction of neuronal assemblies. Oscillations are separated into frequency bands which have variable definitions in the literature (Colgin, 2016; Jensen et al., 2019). Here these ranges are defined as: delta (1–3 Hz), theta (3–7 Hz), alpha (8–12 Hz), beta (13–25 Hz), and gamma (25–100 Hz) (Colgin, 2016). The oscillations generated by various circuits enable the self-organisation of transient neuronal assemblies that store information and underlie cognitive function (Bartos et al., 2007). Electrophysiological techniques have revealed distinct behavioural regimes for each oscillatory frequency during both wakefulness and sleep (Buzsáki and Draguhn, 2004; Koepsell et al., 2010; Adamantidis et al., 2019).

Although mainly present during non-rapid eye movement (REM) sleep, delta oscillations have inhibitory functions that are important for cognitive processing, particularly filtering out unnecessary and distracting stimuli (Harmony, 2013). Theta oscillations are largely thought to be involved in spatial learning and memory (Buzsáki, 2005; Goyal et al., 2020), but are also involved in non-spatial working memory tasks where the “gating” of oscillations increases synchrony across multiple cortical brain regions (Raghavachari et al., 2001). Increases in cross-regional synchronisation of theta and alpha oscillations are present during the generation of mental imagery (von Stein and Sarnthein, 2000). Local increases of alpha synchronisation are seen during the suppression of stimuli, causing the individual to selectively attend to other stimuli modalities (Foxe and Snyder, 2011; Klimesch, 2012), whereas long-range increases of alpha synchronisation across brain areas enhance information transfer between lower and higher order regions, thereby improving sensory integration (Doesburg et al., 2009).

Beta oscillations are prominent during wakefulness and are involved in maintaining neuronal equilibrium, working memory, sensory information integration, and voluntary movement control (Schmidt et al., 2019). Changes in the power of beta oscillations are important for the processing of working memory, and are thought to allow for the filtering of distractions when encoding and integrating information (Schmidt et al., 2019). While it is not fully understood how beta oscillations control movement, bursting activity is thought to be involved in specifying the movement and in regulating associated errors.

The main generators of gamma oscillations are fast-spiking, parvalbumin-expressing basket cells (Buzsáki and Chrobak, 1995). Two different models have been proposed to describe how these cells generate gamma oscillations: one suggests that gamma oscillations occur as a reaction of interneurons following the spiking of excitatory neurons, and the other proposes that the interneurons fire first, thereby generating rhythmic synaptic inhibition that regulates neuronal spiking in postsynaptic neurons (Buzsáki and Wang, 2012; Sohal, 2016). Gamma oscillations are involved in working memory, sensory and visual responses, and long-term plasticity changes such as the strengthening of synapses (Hopfield, 1995; Jensen et al., 2007; Colgin, 2016). These oscillations have been found to be dysfunctional in a range of neurological disorders (Schnitzler and Gross, 2005; Stephan et al., 2009; Başar et al., 2016).

## Oscillatory Changes After Stroke

**Figure 1:**
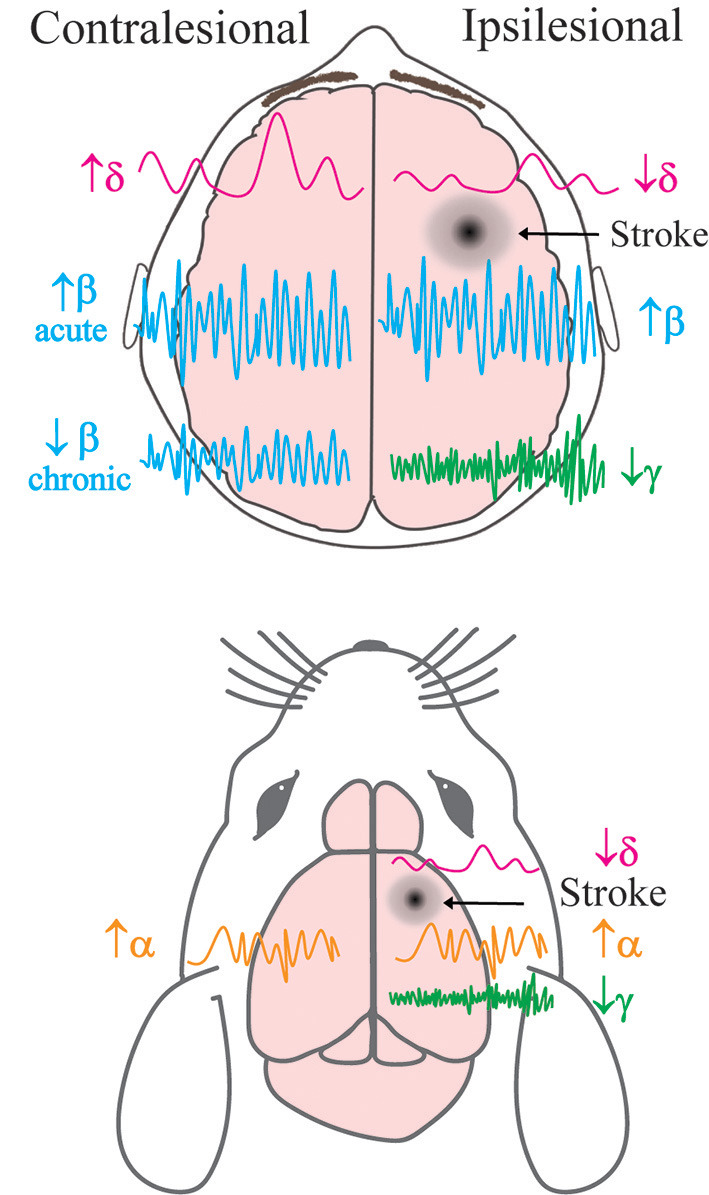
Simplified schema of hemispheric changes to neural oscillatory power following stroke in human and animal models. Previously described alterations to the power of brain oscillations in the contralateral and ipsilesional hemispheres of human patients (top) and mice (bottom) post-stroke. δ = delta, α = alpha, β = beta, γ = gamma frequency bands. Acute refers to <7 days after stroke, and chronic refers to >6 months after stroke.

Many neurological disorders are characterised by changes to oscillatory dynamics, including AD, schizophrenia, autism, and bipolar disease (Matlis et al., 2015; Başar et al., 2016; Jafari et al., 2020), and multiple studies have found changes to brain oscillatory activity following stroke in both humans and experimental animal models (Rabiller et al., 2015) (Figure 1). An increase in low-frequency oscillation power (~1 Hz) is observed in the peri-infarct area within the first week following stroke. This increase can last up to 3 months and correlates with worse clinical outcomes (Laaksonen et al., 2013). Higher delta power in the unaffected hemisphere has also been linked to poorer clinical performance (Tecchio et al., 2007). However, more recent studies have shown that this outcome may be dependent on the stage of stroke, with higher delta coherence (synchrony between regions) predicting worse behavioural recovery in the acute phase after stroke, but better recovery in the chronic phase (Cassidy Jessica et al., 2020). These results show that delta oscillations can be either protective or harmful in recovery processes; the correlation between higher delta power and recovery in chronic stroke suggests that the brain has either adapted to having an increase in these oscillations, or that their effects are only beneficial once a certain amount of recovery has already occurred.

Following stroke there are also changes to behaviour-dependent oscillations that lead to specific impairments depending on the lesion location. Low-frequency oscillations (1.5–4 Hz) in the motor cortex are present during normal functioning and reaching behaviours in mice. After stroke, reaching behaviour is impaired in correlation with a decrease in power of reaching-associated low-frequency oscillations (Ramanathan et al., 2018). The same study found a similar decrease in low-frequency oscillatory power during reaching in a human patient 4 years after stroke. This patient also exhibited associated movement deficits (Ramanathan et al., 2018). These results demonstrate that the reduced power of low-frequency oscillations observed following a stroke to the motor cortex is a key contributing factor to impaired movement. In other human patients with motor deficits, a reduction in movement-dependent low frequency oscillations has been reported in the acute phase; however, motor recovery was observed and oscillatory power returned to normal levels 3 months after stroke (Bönstrup et al., 2019). These results highlight the impact of oscillatory changes after stroke on motor behaviour and show that modulating low frequency oscillations could be the key to improving motor function. The post-stroke increase in delta oscillations observed during rest stands in contrast to the decrease in movement-specific oscillations, suggesting distinct functions throughout recovery.

After stroke, alpha oscillations in human patients are lower in frequency and more synchronised (Petrovic et al., 2017), while desynchronisation of this activity during recovery is associated with improved motor outcome (Westlake et al., 2012; Ray et al., 2020). Alpha oscillatory power has also been shown to be increased in both the unaffected and affected hemispheres in mice 9 days after stroke when compared to controls (Vallone et al., 2016). Similarly, beta oscillatory power is increased in both hemispheres in stroke patients vs. age-matched controls during the acute phase (Assenza et al., 2009). In the chronic phase, higher beta power in the affected hemisphere has been linked to improved motor function, whereas higher beta power in the unaffected hemisphere correlates with worse clinical outcomes (Thibaut et al., 2017). However, an increase in beta coherence between the motor cortex and other regions in the acute phase has also been reported in association with improved outcome 3 months after stroke (Nicolo et al., 2015). Although resting beta power in the sensorimotor cortex may not be changed after stroke, alterations to beta oscillations after motor training could be predictors of improved recovery (Espenhahn et al., 2020). These results suggest that changes to beta oscillations are not consistent across the brain throughout stroke recovery. Gamma oscillations are also disrupted following stroke (Buzsáki and Wang, 2012) and an increase in gamma power in the affected hemisphere has been associated with improved clinical outcomes (Tecchio et al., 2007). Recent research in mice has shown that during the acute phase of stroke, the peri-infarct cortical area exhibits a decrease in power of low gamma oscillations, but no changes in beta or theta oscillatory power (Hazime et al., 2021). Therefore, specifically targeting gamma oscillations in the peri-infarct cortex could be a key target for recovery. Overall, these studies suggest that there are significant changes to neural oscillations post-stroke which also influence recovery outcomes. Further investigation into these oscillatory changes is needed within most frequency bands to explain the differences that are observed between the acute and chronic stages of stroke.

## Tools For Modulating Brain Activity

**Figure 2:**
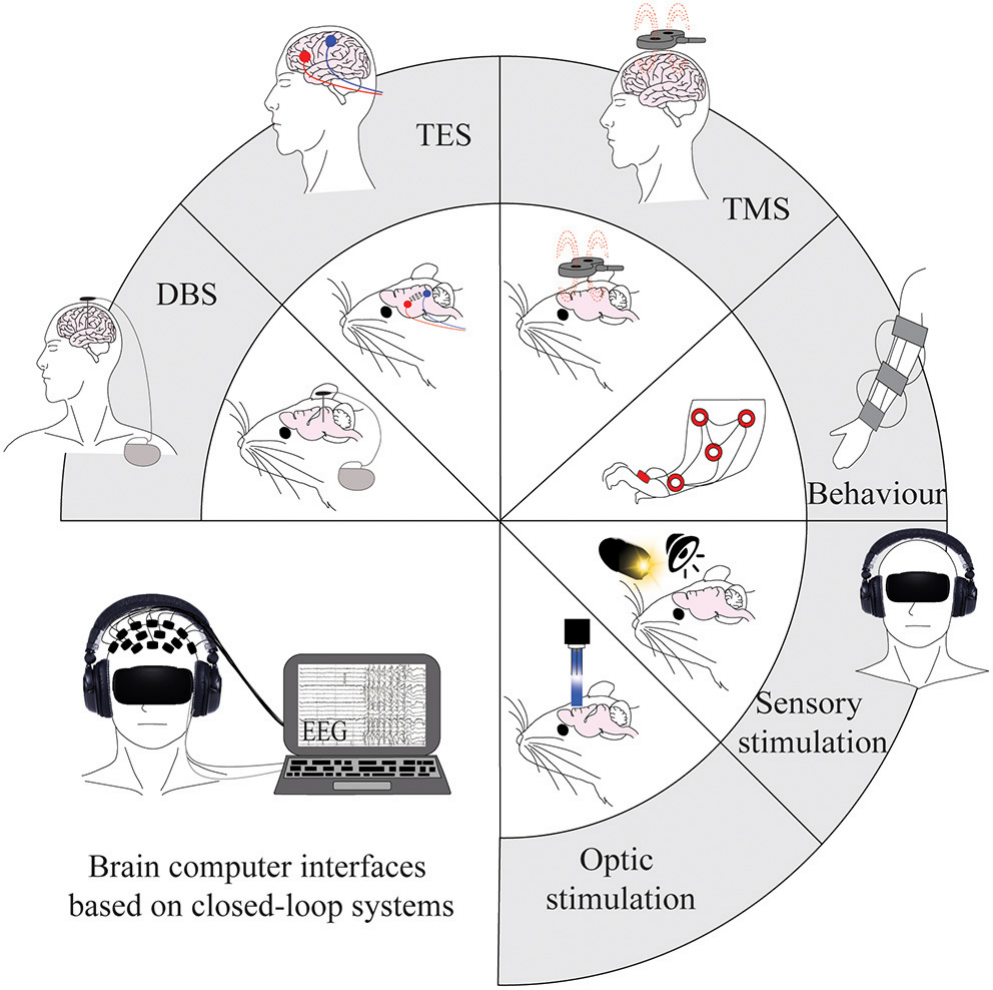
Tools for modulating brain activity post-stroke in human and animal models. Current invasive and non-invasive brain stimulation, sensory stimulation, and optogenetic tools that are used to modulate brain oscillations. Applications are shown on animal models in the inner circle, with the corresponding applications on human patients in the outer part of the circle. The figure in the bottom left represents a combination of current methods and integrates them into a non-invasive closed-loop design that could be considered for future research. DBS, deep brain stimulation; TES, transcranial electric stimulation; TMS, transcranial magnetic stimulation.

Neurostimulation is becoming the prevalent treatment for neurological conditions (Johnson et al., 2013) (Figure 2). However, the ways in which brain stimulation tools, such as electrical stimulation, modulate the brain are not fully understood, and the links are still to be elucidated between molecular changes, circuitry changes, and functional recovery. Despite this, brain stimulation methods have shown potential in motor recovery after stroke (Bao et al., 2020) due to their ability to promote the regeneration of neural connections and plasticity processes.

## Invasive Brain Stimulation

The application of electric stimulation to modulate brain activity has been in use for centuries, and DBS has recently become a routine treatment for diseases with motor impairments (Pycroft et al., 2018). This approach involves implanting electrodes into targeted areas of the brain which are connected to an implantable pulse generator on the skin that delivers chronic electric pulses (Kringelbach et al., 2007). However, the underlying mechanisms of DBS are not fully understood (Anderson and Lenz, 2006; Chiken and Nambu, 2016). Two possible mechanisms are the induction of action potentials via cell activation or the overriding of pathological brain oscillation in the stimulated area (McIntyre et al., 2004; Ashkan et al., 2017).

DBS is effective for diseases which are otherwise treatment resistant, such as PD where it improves motor recovery, decreases beta oscillatory power and increases gamma power (Muthuraman et al., 2020). A recent review showed that DBS is also effective in improving tremors, pain, dystonia, and motor deficits following stroke (Elias et al., 2018). Invasive direct current stimulation has been shown to increase the power of low frequency oscillations in rats after stroke. Stimulation that was applied specifically during a reaching task improved behaviour and increased reaching related oscillatory power (Ramanathan et al., 2018). DBS to the lateral cerebellar nucleus at various frequencies in rats has shown promise in aiding stroke motor recovery by modulating cortical excitability (Baker et al., 2010), and inducing glutamatergic neurogenesis (Chan et al., 2018). However, few studies have investigated how DBS modulates neural oscillations during the stroke recovery process. This treatment can be particularly beneficial for stroke patients with impaired lower limb movement, as the stimulation can reach deep brain areas, including the medial cortex (Franzini et al., 2008). Nonetheless the hazards of DBS need to be taken into account when considering this treatment in stroke recovery, as there is a high chance of an increase in intracranial pressure that may lead to edema, a risk factor for poor clinical outcomes after stroke (Lefaucheur et al., 2013).

## Non-invasive Brain Stimulation

Non-invasive brain stimulation techniques have been extensively used in the treatment of neurological conditions (Johnson et al., 2013). Compared to invasive stimulation, non-invasive techniques avoid the risk of complications during surgery and have a lower risk of causing unintended injuries. In stroke, three methods are increasingly used to promote recovery: tDCS, tACS, and TMS.

tDCS uses low-intensity electrical current flowing unidirectionally from one electrode to the other. The flow of electrons creates a region under the anode where neuronal activity is facilitated and a region under the cathode where activity is inhibited through modifications of transmembrane neuronal potentials and cortical excitability (Tortella et al., 2015). The first use of tDCS to enhance stroke recovery in the acute phase (30 min after stroke induction) was in 2013, and the authors showed reduced lesion volume and improved neurological-severity scores in mice following cathodal stimulation to the sensorimotor cortex (Peruzzotti-Jametti et al., 2013). Anodal tDCS over the target brain area and the cathode placed over the contralateral region, has proved effective in improving both upper and lower limb impairments in stroke patients, as well as anxiety and depressive symptoms (Fusco et al., 2014; Allman et al., 2016; Fleming et al., 2017; Bornheim et al., 2020; Gowan and Hordacre, 2020). Beta coherence between the ipsilesional motor cortex and other brain regions increased in stroke patients who received cathodal tDCS over the contralesional primary motor cortex in the first 4 weeks after stroke, and this increase correlated with improved motor function (Nicolo et al., 2018). Such increased coherence was not seen in the patients treated with intermittent theta burst stimulation, a type of TMS involving bursts of pulses that induce excitability. Anodal tDCS over the ipsilesional primary motor cortex has also been associated with increased alpha coherence, which is thought to be involved in neuroplastic changes and corticospinal excitability (Hordacre et al., 2018). Despite these beneficial effects, the mechanisms underlying tDCS therapy remain unknown. Various studies on animal models have tried to investigate the correlation between brain plasticity responses and phenotypic changes. Recent investigations have shown that tDCS leads to increased brain-derived neurotrophic factor (Bdnf) levels (Podda et al., 2016; Cocco et al., 2020). Other studies have reported effects of tDCS on astrocytes and microglia (Mishima et al., 2019) and shown that it upregulates growth factors including GDF5 and PDGFA, which are associated with increased recovery after stroke (Ahn Sung et al., 2020). tACS is another transcranial electrical stimulation technique that applies oscillatory electrical stimulation which overrides endogenous rhythmic cortical activities during cognitive processes (Antal and Paulus, 2013; Herrmann et al., 2013; Song et al., 2014). Studies using tACS have already shown increases in cerebral blood flow in both hemispheres and lowered resistance in the intracranial vascular bed in patients during the acute phase to 3 months after stroke (Salinet et al., 2014; Wu et al., 2016). Patients in the chronic phase after stroke have also shown network integration and segregation in both motor-related regions and on a whole-brain level after 10 and 20 Hz tACS stimulation (Chen et al., 2021). However, as with tDCS, more research is required to determine the mechanisms underlying the effects of tACS.

TMS uses a magnetic field to induce electric fields in cortical tissue. Electric current flows through a coil generating a magnetic field, that then flows to the neural tissue and generates another electric field (Chail et al., 2018). Repetitive TMS (rTMS) is used in therapeutic interventions and involves long periods of stimulation that are made up of short bursts of pulses. rTMS can either be low frequency (<5 Hz), which causes inhibition, or high frequency stimulation (>5 Hz) which leads to excitation (Valero-Cabré et al., 2017). A recent study has shown that 15 Hz rTMS promotes neuroplastic processes within the brain. Stimulation to the mouse primary motor cortex led to increased dendritic arborisation and spine density of pyramidal neurons in layers 2/3 (Cambiaghi et al., 2021). Most studies into the use of TMS for stroke recovery have focused on treating motor impairments and post-stroke depression (Nowak et al., 2008; Gu and Chang, 2017; Shen et al., 2017; Dionísio et al., 2018). Caglayan et al. showed recovery in mice that received 20 Hz rTMS to the ipsilesional primary motor cortex in the acute phase following stroke, with improvements including increased blood flow, reduced lesion volume, decreased inflammation, and better functional recovery (Caglayan et al., 2019). However, the oscillatory patterns evoked following TMS have not been studied and may underlie the observed recovery. Pellicciari et al. found that low-frequency TMS applied over the primary motor cortex evoked lower alpha and beta power in both hemispheres, and decreased delta oscillations in the contralesional hemisphere, in stroke patients compared to controls (Pellicciari et al., 2018). However, following TMS to the ipsilesional motor cortex, increased cortical responsivity in the affected hemisphere was observed 6-months after stroke. The stroke patients who presented higher TMS-evoked alpha oscillations in the affected hemisphere at baseline also had better functional recovery. The stimulation parameters used in therapy need further investigation and may also need to be personalised, as the degree of disruption to oscillations may impact stimulation parameters.

Comparing results between studies and inferring mechanisms is particularly challenging due to the lack of consistency in stimulation parameters and high injury variability (Fisicaro et al., 2019; Yuan et al., 2020). Additional studies are needed to adequately compare the effects of a wider range of stimulation parameters to determine parameter regimes that promote optimal recovery according to individual characteristics. Therefore, highly adaptive protocols may be necessary to provide the most efficacious treatment.

## Environmental Stimulation

Sensory stimulation, including visual and auditory (or combined) signals, can evoke brain oscillations at certain frequencies in rodent models and humans (Martorell et al., 2019; Zheng et al., 2020). In a mouse model of AD, visual flickering at 40 Hz has been shown to entrain gamma oscillations, leading to decreased synapse loss, microglial inflammation, and amyloid build up, as well as improved cognitive performance (Iaccarino et al., 2016; Adaikkan et al., 2019). Environmental stimulation techniques have also been implemented to improve motor recovery after stroke in animals, using reaching tasks, running wheels, and social enrichment, and in patients, using repetitive upper-limb training, treadmill-supported exercises, robotic arms, and virtual reality (Takeuchi and Izumi, 2013; McDonald et al., 2018). Sensory stimulation of the whiskers at 5 Hz immediately after stroke in rats prevented the formation of an ischemic infarct (Lay et al., 2010; Lay and Frostig, 2014). However, 4 Hz whisker stimulation in a mouse stroke model did not produce any beneficial effect during the acute phase after stroke (Balbi et al., 2021). These contrasting results could be explained by the difference in collateral vascularisation between the two models. Further research is needed to investigate critical time periods for stimulation in both animal models and humans as this may differ across species or even across individuals.

## Optogenetic Stimulation

Optogenetic approaches to stroke recovery use targeted light pulses to activate genetically encoded light-sensitive channels such as channelrhodopsin 2 (ChR2) in order to activate or inhibit specific cell types and circuits with high temporal precision (Lin, 2011). The frequency of the light pulses used is highly dependent on which neurons are being targeted, as this will likely be influenced by their natural firing frequency.

Several studies have already demonstrated the beneficial effects of modulating brain activity with optogenetic tools as a treatment for neurological disorders (Adaikkan et al., 2019). Stimulation of thalamocortical axons at 5 Hz from 3 days to 6 weeks following stroke to the somatosensory cortex in mice increased synaptic bouton formation, and forepaw functioning, however, did not alter blood flow (Tennant et al., 2017). Ten hertz stimulation to the rat contralesional corticospinal tract within the first 14 days after stroke improved motor function (Wahl et al., 2017). Similarly, 10 Hz stimulation to the ipsilesional primary motor cortex in mice within the first 14 days post-stroke led to improved blood flow, the expression of several neurotrophic factors and functional recovery (Cheng et al., 2014). Neurogenesis in the peri-infarct cortex and reduced infarct volume has been seen following 20 Hz inhibition of striatal neurons during the acute phase following stroke in mice, which was coupled with improved performance in the open field and rotarod tests (He et al., 2017). A recent investigation also reported that gamma frequency stimulation of inhibitory neurons in the acute phase of stroke is neuroprotective. In this study the mice showed increased local field potential power, improved cerebral blood flow, decreased lesion volume and brain swelling in the cortex, as well as enhanced motor performance after ipsilateral 40 Hz stimulation within 1 h after stroke (Balbi et al., 2021). Spreading depolarizing waves are common in the acute phase after stroke and are correlated with an increase in lesion volume (Hartings et al., 2016). The occurrence of these waves was decreased during 40 Hz stimulation in Balbi et al. (2021), possibly influencing the decrease in lesion volume observed. Interestingly, 40 Hz stimulation on the contralateral side led to improved motor function after stroke, which supports the hypothesis that after such an event there is an imbalance of excitation and inhibition within hemispheres that, when modulated, can restore function. However, the exact mechanism behind the observed neuroprotective effects remains to be clarified. Studies on mouse models of AD observed changes in microglial morphology after environmental 40 Hz stimulation (Iaccarino et al., 2016), which was not observed during stroke recovery following 40 Hz optogenetic stimulation. Although the potential for targeting gamma oscillations as a recovery tool is increasingly understood, further evidence is needed to support their role in stroke recovery. A recent study in mice has also shown that optogenetic stimulation of pyramidal neurons at 1 Hz during sleep improves functional recovery and increases axonal sprouting (Facchin et al., 2020). However, these beneficial effects were only observed when the stimulation was induced from 5 days post-stroke and not earlier, further suggesting that different oscillatory frequencies may be important for recovery at different time points.

## Future Directions For Stroke Recovery

There is a clear need for further development of non-invasive treatment options for stroke patients that can be applied within a short time window. However, little is known about how non-invasive stimulation methods alter brain oscillations and may promote stroke recovery. New research points to low-intensity focused ultrasound (LIFU) as a promising non-invasive method that is able to target deeper tissue with high spatial resolution (Baek et al., 2020). When LIFU was applied after stroke in mice, the animals exhibited improved motor performance as well as a decrease in the pathological delta power imbalance between hemispheres. Although new methods are being developed, it is also important to refine and integrate current methods that have demonstrated initial promise. A recent study in rats showed that combining tDCS with peripheral sensory stimulation led to the restoration of oscillatory ratio dynamics (Yu-Hang et al., 2017). Therefore, it is likely that the effects of environmental stimulation methods can be enhanced using direct brain stimulation to optimise the restoration of oscillatory dynamics (Figure 2). Optogenetic tools also have the potential to provide more information about cell- and circuit-specific processes that can then be used to develop translatable treatments. Newly developed techniques include the use of red-shifted light-sensitive dyes to increase light penetration depth (Entcheva, 2013; Joshi et al., 2020), bioluminescence to fuse opsins with carrier proteins (Boyle et al., 2015; Jiang et al., 2018), and opsins co-expressed with sonoluminescence or X-ray-inducible nanophosphors (Boyle et al., 2018).

Most studies use open-loop paradigms whereby brain stimulation tools are applied with predetermined stimulation parameters for set durations. Although these can effectively induce oscillations, another approach to enhance endogenous oscillations is via a closed-loop paradigm that uses external stimuli to provide feedback to the subject on the levels of desired cortical activity (Kanta et al., 2019). The use of DBS in a closed-loop system has been investigated in the treatment of AD (Senova et al., 2018) and PD (Fleming et al., 2020) and allows changes to stimulation parameters based upon individual brain activity. Combinatory systems such as EEG-tACS or EEG-TMS can also provide temporal resolution within milliseconds (Raco et al., 2016; ten Oever et al., 2016); however, the stimulation creates artefacts in the EEG recording that need to be corrected for in real time (Noury et al., 2016). In the field of stroke recovery, there is a need for the implementation of these closed-loop systems to modulate oscillatory activity and reduce the high variation in treatment efficacy seen between patients. The recently developed “Embodied Brain” closed-loop simulation model may help us to understand the impacts of specific changes to neuronal circuits and oscillatory dynamics before implementing them experimentally (Allegra Mascaro et al., 2020). Future research should combine environmental and direct brain stimulation techniques in animal models to improve our understanding of evoked and enhanced oscillatory dynamics in order to refine the closed-loop systems used in human patients (Figure 2). The ultimate goal of this strategy is to develop low-risk methods that can be easily used on patients within hours of stroke.

## Concluding Remarks

Increasing evidence shows an association between the modulation of brain oscillations and their potential for neuroprotection. Environmental and brain stimulation methods are successfully being implemented to measure and modulate brain oscillations at different frequencies. The modulation of gamma oscillations within the brain using these stimulation techniques is a promising approach for recovery after stroke. The next major challenge in stroke recovery research will be to translate preclinical data and methods into clinical trials and practical treatments for patients. However, more research with animal models is first needed to mechanistically understand and optimise different treatment methods, with a particular focus on investigating techniques that modulate natural oscillatory patterns. This research should include measuring changes of both cortical and subcortical neuronal activity, neurogenesis markers, interhemispheric connectivity, and communication following modulation.

## Author Contributions

All authors listed have made a substantial, direct and intellectual contribution to the work, and approved it for publication.

## Conflict of Interest

The authors declare that the research was conducted in the absence of any commercial or financial relationships that could be construed as a potential conflict of interest.
